# Gut Microbiota Interact With the Brain Through Systemic Chronic Inflammation: Implications on Neuroinflammation, Neurodegeneration, and Aging

**DOI:** 10.3389/fimmu.2022.796288

**Published:** 2022-04-07

**Authors:** Yi Mou, Yu Du, Lixing Zhou, Jirong Yue, Xianliang Hu, Yixin Liu, Sao Chen, Xiufang Lin, Gongchang Zhang, Hengyi Xiao, Birong Dong

**Affiliations:** ^1^ Geroscience and Chronic Disease Department, The Eighth Municipal Hospital for the People, Chengdu, China; ^2^ Department of Emergency and Critical Care Medicine, The Fourth West China Hospital, Sichuan University, Chengdu, China; ^3^ National Clinical Research Center for Geriatrics, West China Hospital, Sichuan University, Chengdu, China

**Keywords:** gut microbiota, intestine barrier, systemic chronic inflammation, blood–brain barrier, neuroinflammation and neurodegeneration

## Abstract

It has been noticed in recent years that the unfavorable effects of the gut microbiota could exhaust host vigor and life, yet knowledge and theory are just beginning to be established. Increasing documentation suggests that the microbiota–gut–brain axis not only impacts brain cognition and psychiatric symptoms but also precipitates neurodegenerative diseases, such as Alzheimer’s disease (AD), Parkinson’s disease (PD), and multiple sclerosis (MS). How the blood–brain barrier (BBB), a machinery protecting the central nervous system (CNS) from the systemic circulation, allows the risky factors derived from the gut to be translocated into the brain seems paradoxical. For the unique anatomical, histological, and immunological properties underpinning its permeable dynamics, the BBB has been regarded as a biomarker associated with neural pathogenesis. The BBB permeability of mice and rats caused by GM dysbiosis raises the question of how the GM and its metabolites change BBB permeability and causes the brain pathophysiology of neuroinflammation and neurodegeneration (NF&ND) and brain aging, a pivotal multidisciplinary field tightly associated with immune and chronic systemic inflammation. If not all, gut microbiota-induced systemic chronic inflammation (GM-SCI) mainly refers to excessive gut inflammation caused by gut mucosal immunity dysregulation, which is often influenced by dietary components and age, is produced at the interface of the intestinal barrier (IB) or exacerbated after IB disruption, initiates various common chronic diseases along its dispersal routes, and eventually impairs BBB integrity to cause NF&ND and brain aging. To illustrate the immune roles of the BBB in pathophysiology affected by inflammatory or “leaky” IB resulting from GM and their metabolites, we reviewed the selected publications, including the role of the BBB as the immune barrier, systemic chronic inflammation and inflammation influences on BBB permeability, NF&ND, and brain aging. To add depth to the bridging role of systemic chronic inflammation, a plausible mechanism indispensable for BBB corruption was highlighted; namely, BBB maintenance cues are affected by inflammatory cytokines, which may help to understand how GM and its metabolites play a major role in NF&ND and aging.

## 1 Introduction

Multiple basic and clinical studies support the opinion that the gut microbiota (GM) impacts the health and diseases of the brain (1–5), with a special focus on BBB (2, 3). Nevertheless, few neural diseases caused by GM have been recognized in clinics. Coincidentally, Miller Fisher’s syndrome, a subtype of Guillain–Barre’s syndrome, is caused by cross-reaction with antibodies targeted to mural glycoconjugates of *Campylobacter jejuni*, which share similar epitopes with human ganglioside GQ1b (6). *C. jejuni* is a conditional colonization species in the human gut (6). For another example, schizophrenia and its subtype are associated with several enterobacterial strains, such as *Streptococcus vestibulitis* (7). A couple of reports suggested that minocycline is efficient in deficit schizophrenia treatment as an adjunct therapy (8, 9). Although the underlying pharmaceutical mechanism is still controversial, abnormal GM and serum proinflammatory factors have been implicated as contributing factors for brain astroglial activation (9).

**Figure 1:**
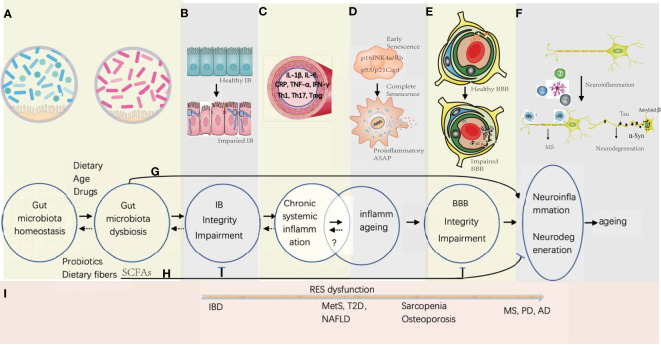
Gut microbiota-associated processes of neuroinflammation and neurodegeneration in aging. **(A)** Gut microbiota homeostasis could be disturbed by Western diet, drugs, and increasing age, while the probiotics and dietary fiber is beneficial in maintaining the homeostasis. **(B)** Gut microbiota dysbiosis would induce the gut epithelial inflammation together with the TJ disintegration and the apoptosis of the gut epithelial cells. **(C)** Proinflammation factors and primed immune cells from the gut tract would translocate into systemic circulation, which might be reversible by promptly correcting the gut microbiota homeostasis. **(D)** Senescence of somatic cells could be induced by unresolved chronic systemic inflammation with activation of signaling pathways of p16INK4a/Rb and p53/p21Cip1 at early stage, and progressed into full senescent stage by secreting proinflammatory ASAP. Whether the stage of the inflammaging is reversible is under investigation. **(E)** Sustained systemic inflammation would cause the remodeling of the BBB architecture with increasing permeability and collapse. **(F)** Influx of the immune cells and proinflammatory factors would activate the microglia in the brain, and further stimulate neuroinflammation and neurodegeneration. **(G)** Before the deficiency of IB and BBB, some small molecules derived from the metabolites of the pathogenic gut symbiotics would be transported into the brain and induce CNS inflammation. **(H)** SCFAs produced by probiotics have the potential to enhance the integrity of IB and BBB, and decreases the risk of neuroinflammation and neurodegeneration. **(I)** In general, with the dysfunction of the reticular endothelial system (RES), local gut inflammation induced by microbial dysbiosis would spread to other organs gradually to cause different diseases from IBD of gut to MS, PD, and AD in the brain.

The mammalian brain is the immune-privileged organ protected inside the skull, wrapped within the meninges, separated from blood with blood–brain barrier (BBB) and blood–cerebrospinal fluid (CSF) barrier, and floated as a whole in the CSF with the segregation of leptomeninges (10). Recent advances strongly suggested the correlation between the GM and neuroinflammation & neurodegeneration (NF&ND), but how the risk factors from the gut are transmitted into the brain would be the key to understanding NF&ND and aging. According to the gut–brain axis, there are three pathways through which the GM can interact with the brain, including the endocrine, neural, and immune pathways. Here, we mainly focused on the immune pathway *via* systemic circulation because the BBB is not only an immune barrier to separate peripheral inflammatory factors and maintain CNS immune privilege but also an interface influenced by the distal abdominal viscera at a distance. In the event of sustained, low-grade, systemic chronic inflammation, the healthy BBB is a delicate dynamic structure that maintains brain homeostasis with the ability to resist inflammation (10). Supposing that chronic inflammation were mainly elicited by gut microbiota, the occurrence of NF&ND is a multifactor and multistage process that requires the dysregulation of both IB and BBB (**Figure 1**). More severely, clinical investigations reported that barrier damage of both the intestine and brain with gut microbe infiltration was found in deficit schizophrenia patients (11), which suggested that barrier breach might serve as a biomarker for the initation and progression of the diseases.

For aging, BBB disruption increases the influx of neurotoxic pathogens into the brain parenchyma and the neurodegeneration became rather complicated, including [i.e. AD, PD, and metabolic syndromes (MetS)] became rather complicated, together with the gut microbiota-induced chronic systemic inflammation (12, 13). For example, MS incidence in aging population is increasing globally and present a unique pathophysiological transition from an inflammatory to a neurodegenerative profiles (14). Preclinical studies strongly suggested the influence of GM composition on MS (15–17). Moreover, the relations among the gut microbiota, intestinal barrier permeability, and brain pathogenesis had been reviewed (18). But the close relationship between BBB and GM, especially GM-induced chronic inflammation, needs more inquiries.

Although multiple studies provide important clues for gut microbiota-induced BBB breakage, an integrative and precise outlook to understand why and how the BBB is dyregulated in the condition of gut-induced SCI from a mechanistically biological perspective is still lacking. In this review, we first summarize BBB components and their relationship with immunity, then provide the BBB structural disruption on NF&ND in response to systemic chronic inflammation and inflammaging together with a particular interest on whether BBB leakage is the hallmark of NF&ND or not; afterwards, we discuss how the neurovascular unit (NVU) senses and responds to the peripheral inflammation and subsequent dysfunction and collapse; finally, we outline the selected important advances about GM influence on NF&ND and aging (**Figure 1**).

## 2 The Roles of the Blood–Brain Barrier in Immunity

The adult brain is composed of 100 billion neurons together with 1 trillion glial cells, accounting for 2% of the body weight and consuming 20% of the energy supply. Three brain blood vessel parameters are conducive to understanding their exchange capacity: 644 km in total length, 20 m^2^ of total vascular surface area, and 40 µm of mean distance between any capillary vessels (19). BBB formation and maintenance depend on important growth factors and morphogens; see reviews (20). Paradoxically, the brain is an immune-privileged organ, and yet active immune surveillance also takes place in the brain, whose dynamics rely on the ingenious structure of the BBB to maintain homeostasis. The functional unit of the BBB is called the NVU, whose coupling is key to the permeability of the BBB. The NVU is composed of brain endothelial cells (BECs), pericytes, a basement membrane (BM) layer, and astrocytes (19, 20). Members of the BBB work synergistically to maintain the healthy brain (NVU coupling), while proinflammatory factors and lipopolysaccharide (LPS) impair BBB integrity (NVU uncoupling); see sections 4.2 and 4.3. In the condition of uncoupling, influx of neurotoxic components induces the most dramatic consequences related to NF&ND, a hallmark tightly associated with the aging process (21). Therefore, we summarize the major histological components of the NVU and their roles as the immune barrier.

### 2.1 Brain Endothelial Cells and Their Immunity

BECs have significantly low penetrance to the intravascular materials, in contrast to their counterparts in the peripheral tissue and organs. A thick luminal glycocalyx layer, special TJ structure, lack of fenestration, and highly selective influx and efflux transporters of BECs underpin the trans-endothelial permeability and guarantee metabolic and immunological homeostasis for normal brain functions (22). The paracellular permeability of BECs mainly depends on tight junctions (TJs) and adherens junctions (AJs) (23). Recent progress suggested that the vimentin network, a kind of intermediate filament, was found to break apart under disease conditions concurrent with stress fiber formation and VE-cadherin disintegration that could change BBB permeability (24), which provides an example of how the intracellular cytoskeleton of BECs is involved in permeability regulation. Moreover, the specialized transcription program of BECs was retrograded to that of common endothelial cells by various pathogenetic conditions (25). The transcription program included a panel of 136 genes that are essential for BBB features, including molecules of Selectin E and P with the ability to regulate leukocyte trafficking (25). Hence, the peri-endothelial and trans-endothelial permeability of BECs would be injured under pathological conditions.

On the other hand, BECs are not the passive front line to protect the immune privilege of the brain. Under healthy conditions, luminal surface expression of the molecules for leukocyte adhesion and transmigration is low (see section 4.2). Astrocyte-secreted SHH interacts with BBB endothelial cells, which is the major force to maintain BBB integrity and immune quiescence (26). In the event of cerebral ischemia, it has been reported that injured BECs expressing CCL5 recruit circulating regulatory T cells (Tregs) to repress local inflammation by secreting PD-L1 and matrix metallopeptidase (MMP) 9 (27). An earlier report suggested that the expansion of Tregs would ameliorate pathogenic T cell (TC) infiltration into the CNS (28). However, another report suggested that CCL5 can also induce Th17 cell transmigration across BECs *in vitro* (29). Together, the pleiotropic effects of BECs on BBB integrity need further in-depth investigation.

### 2.2 Astrocyte and Glia Limitans

The function of NVU astrocytes is versatile, supporting the vascular endothelium, reacting to immune stimuli, forming endfeet and glia limitans, and regulating intracerebral liquid flow. Astrocyte endfeet together with their secreted BM enclose the brain microvessels seamlessly to form a glial limiting membrane, namely, the glia limitans. In response to IL-1β, astrocytes enhance the tightness of their endfeet by expressing TJ components (CLDN-1 and CLDN-4), while conditional knockout mice (Gfap-Cre Cldn4fl/fl) showed peripheral T lymphocyte infiltration, such as CD4+, CD11b+, and CD45+ TCs (30). This result demonstrated that glia limitans is an immune barrier that blocks the entrance of TCs into the parenchyma (30). Connexin 43 (Cx43) of astrocytes participates in the maintenance of BBB integrity, and the loss of astrocyte Cx43 causes continuous immune cell recruitment (see reviews) (31).

Because of the unique position of NVU astrocytes, they can crosstalk with neurons inside the brain (32) or receive cytokine signals from the peripheral circulation (33), followed by chemokine release, such as CCL6 (32), to activate lymphocyte trans-endothelial migration. Under pathological conditions, miscellaneous chemokines can be secreted from astrocytes, including CXCL1, to mobilize neutrophils *via* the CXCL1–CXCR2 axis (34); astrocytes can secrete MCP-1, IP-10, and IL-6 by interacting with monocytes in the presence of IFN-alpha and imiquimod (35). In addition, the MCP-1/CCR2 axis is an important chemoattractive pathway for circulating monocytes. Another astrocytic secreted chemokine, CXCL12, whose expression is regulated by TNF (36), could restrain CD4+ leukocytes in the perivascular space *via* CXCL12-CCR4 chemotaxis (37). TNF can be produced during inside the brain, but it might also be transported from the circulation outside peripherally (38) (**Figure 1C**).

According to an experimental acute viral encephalitis model of CCL2(-/-) knockout mice, CCL2 is the key chemotactic factor that facilitates monocyte migration through glia limitans from the perivascular space (39). CCL2(-/-) knockout did not significantly influence the penetrance of CD4+ and CD8+ cells, but compromised CNS immunity and viral clearance and delayed leukocyte transmigration for 5 days (39). Moreover, neuroinflammation from the abluminal side of BECs caused BBB disruption and increased leukocyte infiltration into the CNS (40). Implicitly, these studies strongly uphold that NVU astrocytes and their endfeet are dynamic regulatable immune machinery.

### 2.3 Pericyte Effect on NVU Integrity and Immunity

Brain pericytes are heterogeneous mural cells attached to the capillary endothelial vasculature in the CNS; see reviews (41). The function of pericytes is still under investigation, but their roles in BBB formation and maintenance have been recognized, including epithelium integrity, astrocytic endfeet, leukocyte trafficking, and vascular immune homeostasis (42). Pericytes are embedded in BM and directly attach to endothelial cells (ECs) by a special “peg-and-socket” ultrastructure (43). A recent 3D electron microscopic technique provides a distinct peg-shape projection between pericytes and endothelial cells (44). Noticeably, the peg abundance is different between pericytes, but the ratio of pericyte pegs and endothelial pegs is conserved (44). Functional studies suggested that pericytes regulate astrocytic endfeet and BBB endothelium formation. Rather than disintegration of TJs of BECs, the increased transcytosis seems to be the major contributing factor to change the regional BBB permeability in the event of pericyte loss (45).

*In vitro* transmigration experiments showed that the monocyte transmigration rate could be mitigated by pericytes (46). *In vivo*, pericytes restrict lymphocyte transmigration into the brain parenchyma (42). Under conditions of pericyte deficiency, abnormal regional BBB permeability (45) and dysregulated leukocyte trafficking (42) in the CNS have been identified. Moreover, the leukocyte trafficking frequency of adult pericyte-deficient mice [Pdgfb (ret/ret)] is negatively associated with vessel pericyte coverage, and the simulation of viral infection of encephalomyelitis is lethal to Pdgfb (ret/ret) mice caused by a massive influx of immune cells (42). Loss of pericyte-secreted laminin enhances BBB permeability and compromises aquaporin 4 (AQP4) expression (47). Therefore, the essential characteristics of pericytes include the maintenance of BBB permeability, and loss of pericytes indicates the uncoupling of NVU integrity.

### 2.4 The Roles of the Basement Membrane in Immunity

The BM of the BBB is a multilayered, extracellular matrix composed of laminin, collagen IV, nidogen, and proteoglycan, which is produced by the interplay between astrocytes, BECs, and pericytes (22, 47). Briefly, the functional role of most BM members has been examined by knockout mouse studies; BM not only allows fluid and soluble molecule passage but also provides versatile functionality, such as blocking leukocyte infiltration and binding growth factors (see reviews) (22). By postmortem diagnostics, a case–control study suggested that increased collagen type IV expression and a decreased lumen/wall ratio were found in elderly people who died of noncerebral causes (48).

BM laminins influence T lymphocyte extravasation and migration into the brain parenchyma. Laminin alpha4/5 participates in T lymphocyte extravasation, but laminin α5 inhibits T-cell migration by directly binding to α6β1 receptors (49, 50). In 2020, Zhang et al. suggested that brain endothelial-secreted laminin 511 keeps TCs immovable and inhibits their differentiation to Th17 cells by binding the α6β1 or αvβ1 integrin receptors on TCs (51). Interestingly, it has been reported that laminin 511 of BM and endothelial cells are necessary for monocyte differentiation into macrophages (50), a mechanism that may play a role in the NVU, where a similar induction postmortem microenvironment exists for circulating monocytes. Laminin 411, a BM member expressed in health and diseases, also binds MCAM (CD146), a human marker for circulating Th17 cells, and facilitates Th17 cell transmigration into the brain (52). However, the interaction between Tregs and pathogenic Th17 cells may also occur at the luminal surface of BECs (section 2.2), and few reports have studied the role of Tregs in the BM of the BBB.

### 2.5 Perivascular Space

The perivascular space (PVS) is located between the abluminal BM secreted by BECs and astrocyte-secreted BM, and both are aligned in parallel (37, 53). Of note, PVS is a part of the highly organized glymphatic system sharing key functions with the lymphatic vessels of peripheral tissues (54, 55). The concept of glymphatic innovates a previous conjecture that brain homeostasis depends on the classical cellular protein degradation pathway to maintain the protein balance between synthesis and clearance (55). *In vivo* experiments demonstrated that the fluid and waste clearance of the brain could also be removed by PVS. Both the glymphatic and lymphatic systems are drained into the venous system to join the systemic circulation (54). Few reports have suggested that perivascular drainage of solutes is impaired in the aging mouse brain and in the presence of cerebral amyloid angiopathy (56). Accordingly, the crucial role of PVS in brain immune surveillance, NF&ND, and brain aging would be a promising field.

In healthy individuals, macrophages can be found in the PVS, whose expression of MHC II molecules indicates their ability to present antigens to activate infiltrating immune cells. The restimulation of the lymphocytes from the blood flow would grant their chances to enter the parenchyma (53). In spared nerve injury (SNI) mice, a persistent increase in macrophages in the PVS was mediated by CXCL12-CXCR4 signaling (57). The importance of the SNI model implied that the overexcited neurons induced by pain would probably activate brain microglia and astrocytes, followed by chemokine secretion, because lymphocytes migrate along the gradient of the chemotactic concentration. More importantly, peripheral inflammation also enhanced the opportunities for monocytes to turn up in PVS, which was demonstrated by intravenous CXCL12 administration (57). Under disease conditions, a subset of CD14+ monocytes can differentiate into perivascular CD83+CD209+ DCs after the transmigration of the inflamed BBB, which can further induce the naïve CD4+ into IL-17 secreting TCs (58). Collectively, perivascular immune cells are in a standby mode that is actively engaged in immune surveillance and quickly responds to challenges as the first wave of inflammatory cells; if pathogens are not cleared successfully, local inflammation would recruit more immune cells and transmigrate into the CNS.

## 3 Chronic Inflammation, BBB Disruption, and NF&ND

### 3.1 Systemic Chronic Inflammation and Inflammaging

In ancient times, inflammation was described as redness, warmth, pain, and functional loss (59). Currently, inflammation is the host response to eliminate xenobiotics and unrecognized endogenous signals to the immune system (59). Successful clearance of pathogens follows the resolution of the inflammation, but unresolved acute inflammation would cause disease aggravation or turn out to be chronic inflammation with serum proinflammatory biomarkers such as IL-1β (60), IL-6 (60), TNF-α (60), C-reactive protein (CRP) (60), or IFN-γ (**Figure 1C**).

To date, the pathophysiology of the chronic inflammation profile is more complicated than we had expected. Epidemiological studies have demonstrated that chronic inflammation is closely associated with chronic diseases, cancers, and aging. Mechanistic studies suggested that chronic inflammation causes slow tissue remodeling and organ dysfunction accompanied by autoimmune responses and cellular senescence, which might constitute the common pathogenic changes of various chronic diseases. Researchers from different fields might use different terms to describe the subject rather than chronic inflammation, such as SCI, low-grade systemic inflammation, low-level systemic inflammation (LLSI) (61), chronic low-grade inflammatory phenotype (62), inflammaging (63), and immunosenescence (64). Here, we prefer to use chronic inflammation and SCI to describe the continuous, low-grade, systemic sterile inflammation status.

Generally, SCI and inflammaging can be used interchangeably, but there remain several important points to distinguish SCI from inflammaging (**Figures 1C, D**). SCI is sustained immune activation with the inability to remove pathogens, which may be caused by the presence of certain ineradicable factors, including gut microbiota dysbiosis, chronic infections, and insomnia (60). Both internal and environmental risk factors, including lifestyle factors, could trigger SCI; SCI is associated with multiple chronic and autoimmune diseases, and the early identification and eradication of pathogens would prompt health recovery. While inflammaging refers to a low-grade, nonresolving inflammation status in elderly individuals, it is a common feature of the biological aging process (63). Inflammaging often means a higher proportion of senescent somatic cells in the tissue and organ. Most likely, physiological redundancy for self-repairment and self-regeneration has been weakened, and removal of the impairing factors would not contribute to rehabilitation easily. Indeed, inflammaging is associated with chronic comorbidity, frailty, NF&ND, geriatric syndrome, disability, and premature death. That is, inflammaging would not easily be restored to health even after the removal of pathogens, which is quite different from the incipience of LLSI and SCI. Hence, it is worth assuming that sustained SCI would cause inflammaging, and inflammaging was the common outcome of SCI shared by different pathophysiologies.

Aging influences both cellular and humoral immunity. Macrophages decrease phagocytosis, autophagy, and TLR expression while increasing proinflammatory cytokine secretion (65). The number of circulating CD16+ monocytes increases, while IFN production is diminished (65). Thymic involution is the major reason for T-cell aging, which leads to the release of fewer naïve TCs and more autoimmune TCs into the systemic circulation (66). Under immunosenescence, CD27 and CD28 are downregulated, while the expression of CD57 is elevated (64). Senescent cells become the source of proinflammatory cytokines in the circulation with a special term senescence-associated secretory phenotype (SASP) (**Figure 1D**). Strictly, cellular senescence is a stable cell arrest status unresponsive to mitogenic signals that evolves from highly dynamic, multistep processes of cellular and molecular changes to develop the complex SASP with a flattened morphology that involves the activation of the p53/p21 WAF1/CIP1 and p16INK4A/pRB tumor suppressor pathways (64). SASP is associated with the secretion of proinflammatory cytokines, chemokines, and extracellular matrix enzymes, and JAK/STAT pathway activation is responsible for cytokine release; see review (67). Altogether, SCI and inflammaging could be assumed to be two continuous and overlapping pathogenic processes, and the diagnosis of SCI as early as possible would be beneficial to delay the progression of aging.

### 3.2 BBB Structural Disruption in SCI

To be an exquisite machinery with highly regulatable dynamics, the BBB or NVU is one of the key determinants influencing brain health and diseases (**Figure 1E**). Unresolved SCI is a detrimental factor for BBB integrity (68, 69), and the compromised BBB architechture is a hallmark of many cerebrovascular disorders (20). The structural integrity of BECs is characterized by special junctional complexes, active influx and efflux transporters, low vesicle transcytosis, low leukocyte adhesion molecules (LAMs) expression, and a thick glycocalyx (20). Fascinatingly, proinflammatory cytokines dysregulate cellular junctions and change BBB permeability (69). Here, we mainly summarize the effect of SCI on BBB integrity and the underlying mechanisms (see section 4).

An early *in vitro* transmigration experiment demonstrated that TNF-α enhances endothelial permeability (68). In 2013, it was demonstrated that LPS and TNF-α are damaging factors on the endothelial glycocalyx, which decreases glycocalyx thickness and stiffness (70). Recently, novel advances suggested that BECs reduced their expression of TJs and AJs, CLDN-5, OLDN, and VE-cadherin in response to IL-6 (69). Under the chronic status of stroke, upregulated CLDN-1 interferes with CLDN-5 incorporation into the TJ complex; CLDN-1 is not specific to the BBB, whose presence is concurrent with the endothelial proinflammatory phenotype and postpones the recovery of BBB integrity (71). Alternatively, it is also necessary to determine the role of aberrant BEC expression of CLDN-1 under chronic inflammatory conditions.

In addition, IL-17 and IL-22 can disturb TJs of the BBB *in vitro* and *in vivo* (72). Under GM-induced SCI conditions, interferon-γ, IL-17A, and zonulin can quickly enhance the permeability of the IB and BBB by modulating TJs and their associated cytoskeleton (73). Moreover, instead of IL-17F, the combination of IL-17A and IL-6 changes the transcription of TJ-associated genes and BBB impairment *in vitro* (74). In an experimental autoimmune encephalomyelitis (EAE) mouse model or in bEnd.3 cells, IL-17A amplified the endothelial production of reactive oxygen species (ROS) followed by cellular contractile mechanism activation and CLDN downregulation (75).

Moreover, the transmigration of immune cells would cause more severe damage to the BBB structure (**Figure 1E**). For example, the translocation of the activated Th17 cells across the BBB became frequent and were proven detrimental to the BBB in the event of diseases (72). Th17 cells have the capability to secrete matrix metalloproteinase members 3 and 9 (MMP-3 and MMP-9) with the ability to decompose the BM of the NVU (72). Moreover, MMP-2 and MMP-9 activity become stronger at the parenchymal border, which speeds up peripheral leukocyte migration through the BBB by decomposing the BM and glia limitans (76). In a traumatic brain model, compromised BBB permeability and CCL2 chemotaxis facilitated monocyte penetration through the BBB, which resulted in aberrant emotional activity and temporary memory deficiency (77).

From an integrative perspective, BBB architecture is maintained by multiple intrinsic and extrinsic signaling factors, interwoven with developmental cues and immune responses. In addition to cytokines, many other factors, such as VEGF, HIF-1, and ROS, substantiate the BBB structure dynamics; see reviews (20, 78). Here, we mainly focused on how chronic inflammation induced by environmental factors or lifestyles serves as the starting point to influence brain health and diseases. If necessary, the impact of neuroinflammation on BBB dysfunction would also be described because the NVU receives signals from both sides, the parenchyma and luminal side. A composite pathogenesis paradigm of NF&ND focused on the BBB would also have included genetic predisposition, and most neurodegeneration investigations provide more information between genetic predisposition and disease pathogenesis but less about the BBB breaks down. Nevertheless BBB is modulated in diseases; presumably, strengthening BBB integrity is an attainable way to alleviate the progression and severity of NF&ND.

### 3.3 SCI and NF&ND

Active immune surveillance exists in the brain parenchyma, and even the brain is recognized as the immune privilege site (37, 53, 79). Innate and adaptive immunity scrutinizes the CNS to maintain the homeostasis of neuronal activity. In 2012, Ransohoff & Engelhardt proposed a theory that the adaptive immune responses against CNS antigens are primed in the deep cervical lymph nodes (DCLNs), which collect the CSF drained from cerebral ventricles together with soluble antigens as a way to potentiate NF&ND; see reviews (53). Meanwhile, an increasing number of studies have suggested a strong role of gut microbiota in NF&ND and aging (see sections 5.5 and 6), and the mechanism strives to be established. Although the BBB is a structure that is hard to examine *in vivo*, it is a nonnegligible border that is logically involved in defending inflammation and one of the critical entrances for circulating cytokines and lymphocytes as those of the blood–CSF barrier; see reviews (80). The healthy body normally produces inflammatory factors at low but detectable concentrations, and their overproduction or deregulation is essential for the pathogenesis of many neurological diseases *via* BBB dysregulation. Even in germ-free mice, a small subset of T lymphocytes (CD69+ CD4+) migrate into the CNS and guide microglial maturation, by pruning the excessive neuronal synapse pruning, and the increased gut commensal load enhances the CNS translocation of CD4+ lymphocytes (81). Therefore, it is worth hypothesizing that inflammatory fluctuations in the brain parenchyma and systemic circulation change BBB permeability, followed by an amplified local proinflammatory response at the BBB interface. From a lifelong perspective, if the etiology cannot be found or removed, nonstopping chronic inflammation will continuously modulate BBB architecture and foster the strength of neuroinflammation, a common pathophysiology underlying various forms of neural degeneration.

According to this assumption, we focused on chemokines, immune cells, and microglia to illustrate the pathogenesis of NF&ND (**Figure 1F**). Chemokines are a group of molecules influencing BBB integrity that regulate lymphocyte chemotaxis (82). For instance, BEC expresses CXCL12, CCL19, CCL20, and CCL21 to maintain a homeostatic status ready for CNS immunosurveillance with multiple neural regulatory activity, including neurogenesis and neuronal survival (82). Strikingly, the abundance and types of chemokines from chronic disease patients vary significantly. A cohort over 60 years in Brazil showed serum biomarker profiles of inflammaging, especially for increasing levels of CXCL8, CXCL9, and IL-6 (83). In a German cohort to study the chemokine profiles in CSF among neuroinflammation (NF) patients, the salient cytokine profiles characterized pathophysiology of NF were suggested (84). Briefly, in contrast to non-NF patients, 26 out of 36 chemokines in CSF increased significantly in MS and other NF patients, including CXCL13, CCL3, CCL7, CCL8, and CXCL9, and changes in the remaining 10 molecules (CCL2, CCL24, CCL26, etc.) were not significant; for viral infection, CX3CL1 and CXCL12 increased; for bacterial meningitis, CCL11, CCL13, CXCL1, IL6, IL10, and TNFα increased (84). Moreover, serum CCL2 (monocyte chemoattractant protein-1, MCP-1) expression is elevated in MetS (85), and CCL2 has the potential to activate the p38 MAPK pathway *via* CCR2, which causes BBB breakage (86). Meanwhile, CCL5 is the chemokine that prompts microglial migration to vessels (87). Interesingly, CCL2 and CCL5 are included in the highly elevated serum chemokines of T2D patients (88). Therefore, chemokines also seem to be crucial clues for understanding the effect of gut mucosal immunity on the CNS *via* SCI. For more information about cytokines in NF&ND, see reviews (89). In MetS patients, serum concentrations of IFN-γ, EGF, IL-1α/-1beta/-2/-4/-6/-8/-10, MCP-1, and TNF-α are significantly higher than those of the controls (85). Interestingly, an India cohort study suggested that IL-17 was decreased in MetS patients (90). However, in ulcerative colitis (UC), serum IL-17A increases significantly (91), which provides a premise to influence the distal organs such as CNS (92).

**Figure 2:**
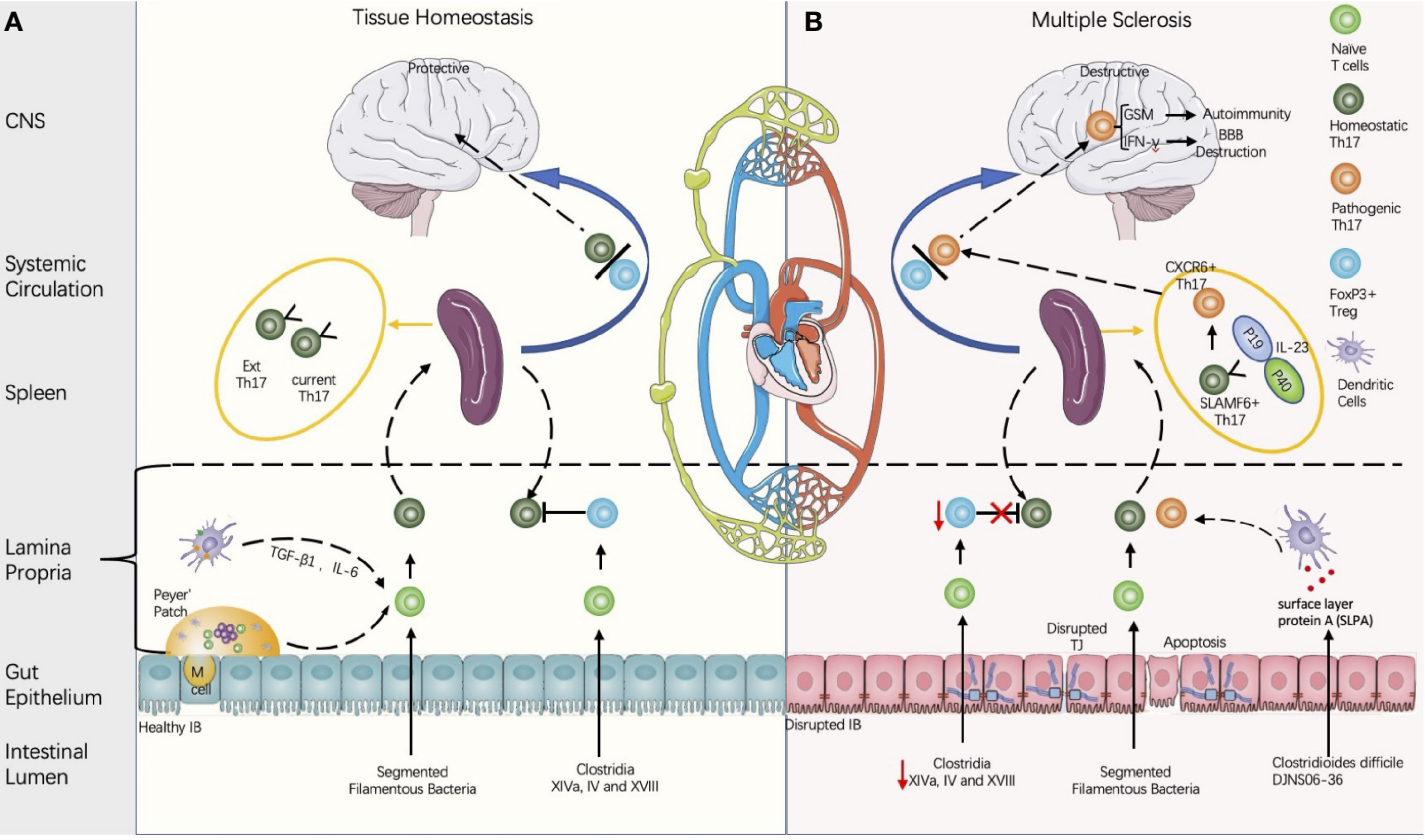
Proposed model of multiple sclerosis initiation derived from the gut microbiota regulated by balance between the Th 17 cells and the regulatory T cells (Th17/Treg). **(A)** Composition of the gut microbiota influences the intestinal barrier (IB) integrity and the Th17/Treg balance. Intestinal Th17 could be induced by segmented filamentous bacteria (SFB), and the contact between the SFB and intestinal epithelial cells is a pre-requirement for Th17 induction (Atarashi in 2015). Meanwhile, CD4+FOXP3+ Treg could be induced either by Clusters XIVa, IV, and XVIII and their 17 strain (93), or by metabolites produced by gut microbiota. Th17 cells is heterogenous and can be classified into current Th17 and Ex Th17. Under homeostatic status, FoxP3+ Tregs restrains the Th17 within protective roles. **(B)** In disease conditions, the decreases of *Clostridium* clusters XIVa and IV are associated with the Th17/Treg imbalance and IB disruption. Some strains of *Clostridioides difficile* contain cross-reactive fragments in their surface layer protein A (SLPA), which is like the epitopes of myelin basic protein (94). *In vitro* experiments demonstrated their potential to induce pathogenic Th17 (94). In the EAE model, stem-like SLAMF6+ Th17 in spleen can be induced by IL-23 and differentiate into CXCR6+ Th17 with homing specificity to the brain; CXCR6+ Th17 is pathogenic by secretion of the proinflammatory factors with potential to destruct BBB (IL-17A, IFN-γ) and to induce autoimmunity (GSM) (95).

The NVU is the major machinery regulating the journey of peripheral leukocytes into the CNS, which could be disrupted by changed chemokines. IL-17 is one of the cytokines with the potential to impair NVU coupling for its ability to recruit circulating neutrophils. IL-17 members can be secreted by Th17 and γδ T cells. Th17 and IL-17 are key factors in MS pathogenesis, and animal models have suggested that the levels of Th17 and IL-17 are tightly associated with the outcome of MS (**Figure 2**). For details on how Th17 and IL-17 activate neuroinflammation, see reviews (92). Interestingly, IgA+ plasma cells derived from the gut could migrate into the brain of MS patients; by secreting IL-10, IgA producing cells could suppress the neuroinflammation of EAE (96). IL-10 is a well-known anti-inflammatory cytokine that can suppress excessive inflammation and hence restrain tissue remodeling and damage (97).

Microglia are the primary innate resident immune cells in the CNS (37) and are derived from primitive hematopoietic progenitors that migrate from the yolk sac during embryogenesis (98). As the principal ROS producer, microglial activation is the hallmark of NF&ND. In 2019, Haruwaka et al. provided an example to explain how microglial activation opens the BBB entrance in response to peripheral inflammation, such as systemic lupus erythematosus (SLE) and endotoxemia, in an experimental mouse model (87). Briefly, in response to systemic inflammation of SLE or induced by peritoneally injected LPS, microglia migrate to the cerebral microvasculature, express CLDN5, and form physical contact with BECs at the beginning (87). However, with extended inflammation, microglia begin to express the phagocytotic marker CD68, whose phagosomes contain the components of astrocyte endfeet AQP4 (87). CCL5 is one of the chemotactic factors guiding microglial migration, and administration of the CCR5 antagonist DAPTA delayed microglial migration and DAPTA can decrease the number of vessel-associated microglia (87). Microglia excitation can be triggered in multiple ways and can stimulate astrocytes to release TNF and glutamate (99). As the resident immune cells in the CNS, microglia interact with neurons to produce chemokines with the capability to recruit leukocytes into the CNS (100). Furthermore, they also engage in crosstalk with CNS-infiltrating CD8+ or CD4+ lymphocytes and other translocated peripheral immune cells under disease conditions (37).

In summary, MS is a good example of chronic systemic inflammation-induced neuroinflammation *via* BBB permeability dysregulation (**Figure 2**). Therefore, future examination of how to traffic immune cells across the BBB could reveal the difference in healthy immune surveillance from NF&ND with diagnostic value (101).

### 3.4 Brain Aging and Inflammaging

As an important indicator of mental decline, brain aging adds another complexity of NF&ND pathogenesis. Meanwhile, both aging and inflammaging have adverse effects on brain function. It is difficult to tell the causal relations between NF&ND and aging, but it is safe to say that aggravated NF&ND is concomitant with accelerated brain aging. A significant transition from the young healthy brain to the age dysfunctional brain involves the hyperactivation of TGFβ signaling in astrocytes (102). Remarkably, dysfunctional microglia lose their neuroprotective properties with age and gradually lead to chronic neurodegeneration (103) (**Figure 1F**).

Microglia activation is the typical feature of NF&ND, which abodes the forthcoming of brain aging (104). First, CX3CR1-mediated microglial migration is compromised, and the decreased motility of microglia is mainly associated with aging instead of disease pathology (103). Second, immune-activated microglia showed strengthened proinflammatory factor transcription (IL-1, IL-6, TNF-α, and IFN-γ) and decreased IL-10 and TGF-β (104); third, microglial cells are sentinels in the CNS to sense pathogens in the surrounding environment (105). Genes associated with sensing ability are changed significantly from endogenous ligand sensing to exogenous microbial ligands in aged microglia; that is, 62% of the upregulated sensing genes were associated with microbial infection, while 81% of genes for endogenous ligand sensing were deregulated (104), which highly suggests a probable cause for the transition of the microglial phenotype from anti-inflammation (M2) to pro-inflammation (M1). Notably, proinflammatory microglia are suggested to cause BBB dysfunction and leakage. Fourth, single-cell sequencing adds depth to the understanding of microglial patterns in NF&ND. In experimental AD mice, there were four functional microglial patterns, including proliferation-, disease-, IFN-, and MHC-II-associated microglial patterns (106). Collectively, microglia in the aged brain create a hostile, oxidative environment for neurons (104), which is consistent with the conception that microglial activation is the greatest risk to brain aging.

Peripheral Th17 lymphocytes appear to be essential in the pathogenesis of numerous inflammatory diseases. Endothelial-associated DCs can stimulate the polarization of CD4+ T lymphocytes to expand the distinct populations of IFN-γ+ Th1 cells and IL-17+ Th17 cells by secreting IL-12p70, TGF-β, and IL-6 (58). An additional study suggested that IFN-γ increases the BBB transmigration frequencies of CD4+ TCs (107) (**Figure 2**). Furthermore, Th17 lymphocytes transmigrate efficiently across BBB-ECs, highly express granzyme B, kill human neurons and promote CNS inflammation through CD4+ lymphocyte recruitment (72) (**Figure 2**).

As the assumed immune privilege organ, the most perplexing question is how NF&ND initiates in the brain, and the answers would be crucial in brain aging prevention. Noe et al. proposed a model of cerebral metabolic syndrome in which BBB dysfunction decreases the exchanges of nutrients, restrains subsequent brain metabolism, and develops NF&ND (23). For example, the expression of the glucose transporter GLUT1 in BECs was decreased in cerebral metabolic syndrome, and energy deficiency in the brain caused neuronal apoptosis and microglial activation (23). The proposal is very illuminating that the incidence of NF&ND could result from oxidative stress and energy metabolism. More interestingly, chronic inflammation is a strong factor influencing GLUT1 and its family member expression (108, 109). Although many facts need further exploration, it is worth assuming that the accumulated minor metabolism disturbance provides a good foundation for the pathogenesis of NF&ND, especially when peripheral SCI begins to infest the CNS vasculature.

### 3.5 BBB Disruption: The Hallmark of Neuroinflammation and Neurodegeneration

The breakdown of the BBB in aging humans and rodents starts from middle age and progresses to the end of the life span (102). BBB has already been regarded as one of the hallmarks of neurological illnesses, whose breakage could be immune-mediated (110). Furthermore, BBB collapse was demonstrated to be an early biomarker of human cognitive dysfunction beyond amyloid and tau (111). Moreover, preclinical investigation suggested that BBB disruption was antecedent to hippocampal atrophy in early AD patients; thus, the clinical view about BBB breakdown possibly occurring before neurodegeneration is proposed (112). Recently, aberrant BBB permeability changes to small molecules, such as water, could lead to clinical-detectable mild cognitive impairment (MCI) (113); of note, the emergence of MCI symptoms did not require BBB permeability larger enough to allow the penetrance of albumin (113). As the pioneering work related to AD diagnosis, the association between BBB permeability and MCI will be examined across the world. Advanced imaging techniques, such as dynamic contrast-enhanced magnetic resonance imaging (MRI) (12) and phase contrast arterial spin tagging MRI (113), have been applied in the detection of BBB permeability in AD, PD, and MS (12); increasing clinical data would help to set up expert consensus and guide practices.

More basically, the association between the BBB permeability changes and NF&ND could have been interpreted by preclinical mechanistic studies. For example, microglia play a central role in NF&ND (see section 3.3). Under brain homeostasis, synergy between neurons and astrocytes activates microglial ramification *via* TGF-β2 signaling and represses the weak inflammatory response of microglia *via* immunomodulatory cues (114). On the other hand, when peripheral inflammation in the bloodstream breaches microvessels (see section 4.3), microglia can transform to a phagocytosis status and remodel neuronal connectivity, which might be one of the molecular mechanisms underlying MCI. Moreover, abnormal oxygen supply and energy metabolism inside the brain might be another way to activate NF&ND with the potential to change BBB permeability. Hence, it is worth assuming that the BBB is a dynamic cellular and molecular organization regulated by the microenvironment in the CNS and the composition in the systemic circulation, and it is reasonable to accept BBB disruption as the hallmark of NF&ND. However, it would be a great effort to determine the BBB permeability of physiology from that of pathology to establish a clinical practicable criterion.

## 4 Chronic Inflammation Changes NVU Signaling

### 4.1 NVU Sensing Peripheral Inflammation

The glycocalyx on the luminal surface of BECs obstructs macromolecule extravasation and leukocyte adhesion and prevents BECs from sensing proinflammatory changes. Hypoxia, inflammation, and TNF-α have been known as disrupting the glycocalyx for some time (115); it is only recently that studies on the impact of systemic inflammation on endothelial glycocalyx damage have been conducted (116). In an experimental endotoxemia mouse model of T2D, LPS administration induced pulmonary edema and serum syndecan-1 elevation (the endothelial glycocalyx injury marker) (116). Nevertheless, acute death of the experimental mice within 48 h may not highlight the natural process of SCI. Of note, the endothelial glycocalyx was impaired before LPS administration in db/db mice for prolonged inflammation (116). Although few direct experimental investigations about how chronic inflammation impairs the BEC glycocalyx have been carried out, it is reasonable to assume that attenuated glycocalyx coats are involved in the early pathogenesis of neuroinflammation and brain aging. The intact glycocalyx may provide higher blocking efficiency by reducing leukocyte extravasation, and the lower level of transmigratory T cells are presented in CNS under healthy conditions.. As the first line to shield SCI, the attrition of the glycocalyx increases the sensitivity of BECs over time.

Lower expression of pattern recognition receptors (PRRs), Toll-like receptors (TLRs), chemokine receptors, and cellular adhesion molecules is another strategy to avoid higher neuroinflammation level (20). In the condition of common chronic diseases and higher age, excessive proinflammatory factors in the bloodstream would change the transcription program of the BECs. In 2010, a study reported that TLR2/TLR3 (117) and CXCR2 (118) expression were enhanced by IL-1beta and TNF-alpha in hCMEC/D3 cells (117). Additionally, immunohistochemistry of brain biopsies from two patients with active multiple sclerosis revealed upregulation of endothelial CXCR2 compared to healthy control tissue (118). Under disease conditions, IL-17 and IL-22 receptors are expressed on BECs in multiple sclerosis lesions (72). The baseline expression of IL-6R in the BBB endothelium *in vivo* and in the cEND cell line is evident, and IL-6 administration elevates IL-6R expression (69). In 2019, Yousef et al. reported that brain endothelial cells presented an inflammatory profile with vascular cell adhesion molecule 1 (VCAM-1) upregulation in aged mice (119); VCAM-1 is one of the key molecules mediating the transmigratory pathway (119). Thus, an increase in membrane expression in response to SCI would strengthen the reactivity of BECs to inflammatory mediators.

At the same time, inflammasome activation is the third defense mechanism that is reinforced by circulating cytokines. Inflammasomes are a group of multiple protein complexes that are downstream effectors of TLRs and PRRs with the ability to sense pathogens in the blood flow, whose activation enhances cytokine maturation or induces cellular apoptosis. Luckily, the expression of TLRs and PRRs could not be detected under healthy conditions (120). However, the major players in SCI (IFN-γ, TNF-α, and IL-1β) can stimulate the transcription of PRRs, and human BECs express quite a few NLRs, such as NOD1/2, NLRC4/5, NLRP1/3/5/9/10/12, and NLRA/X (121), which detect their ligands from the circulation (such as microbial LPS and muramyl dipeptide) to activate the downstream inflammasome pathway in cerebral ECs (121). Moreover, endogenous molecules, such as SAA, aβ, TNF, and HMGB1, could trigger inflammasome activation (122). In the event of high oxidative stress, high fatty acids, elevated triglycerides, and VLDL-cholesterol in the serum, inflammasomes could be activated by pregnane receptors that could sense xenobiotics (123). It has been found that the Nlrp3 inflammasome could downregulate tight junction proteins and increase BBB permeability (124). Logically, inflammasome activation of BECs would have exposed the CNS under stress and stimulated neuroinflammation in the event of SCI, but the precise mechanisms need more inquiries.

In fact, inflammasome mechanisms also exist in other cellular members of the NVU and interact with each other reciprocally in response to the surrounding changes (125). Human cerebral endothelial cells express NLRs and inflammatory components, yet their roles in neuroinflammation have not been fully investigated (121). Furthermore, inflammasomes, which are bridges between adaptive and innate immunity, are expressed in the endothelial (121) and pericyte cells (124) of the BBB. These findings offer a plausible mechanism for how BECs respond to floating pathogens. Collectively, BECs could sense serum SCI, mount local inflammatory responses, and influence nearby pericytes, astrocytes, microglia, and neurons.

### 4.2 NVU Coupling in Brain Homeostasis

Brain homeostasis requires NVU coupling to work synergistically among BECs, pericytes, astrocytes, microglia, and neurons, and uncoupling of the NVU often means influx of neurotoxicants to cause neural pathology. Mouse BECs *in vitro* expressed four cytokines [G-CSF, CCL5, RANTES, and keratinocyte chemoattractant (KC)], while more cytokines were produced in tri-cultures of ECs, pericytes, and astrocytes (126); consequentially, the expression of the junctional proteins and transendothelial electrical resistance (TEER) were enhanced in tri-cultures in contrast to the single EC culture (126). Here, the experiment strongly suggested that the crosstalk among different cytokines is one of the principal influencing aspects of NVU steadiness (126).

BECs remain the frontline and interact with proinflammatory factors and immune cells transmitted by blood flow. Critically, BECs maintain a low proinflammatory profile by interacting with pericytes and astrocytes. For example, it has been reported that one of the cellular adhesion molecules of BECs, CD146, can be repressed after the maturation of pericytes. During mouse brain development, BECs first express CD146 when immature capillaries have no pericyte coverage. Gradually, BECs lose CD146 expression with cerebrovascular maturation, and CD146 can only be detected on pericytes (127). Mechanistically, CD146 is a coreceptor of PDGFRβ with ability to recruit the pericytes, and the recruited pericytes secrete TGF-β1 to suppress the expression of CD146 in BECs (127). Astrocytes can also secrete TGF-β1, whose role is much like the double-edge sword; see reviews (128). In the resting state, by sensing shear strength, BECs secrete deserted hedgehog (DHH) (129) and PDGF-BB to influence astrocytes and pericytes. In contrast to PDGF-BB, DHH and netrin-1, the effector of the Shh signal, drop their expression significantly since the start of inflammation (129).

It appears that astrocytes hide behind the endothelium and BM of the BBB to avoid peripheral proinflammatory influences. Far from the fact, astrocytes actively secrete Sonic Hedgehog (Shh) to fortify the BBB, whose expression level increases with inflammation (130). In the NVU, astrocyte-secreted Shh induces the expression of endothelial netrin-1, and the loss of netrin expression not only disorganizes the formation of TJs but also makes the BBB vulnerable (130). Conversely, astrocytes also respond to BEC-secreted DHH to strengthen astrocytic glia limitans (129); perhaps glia limitans might also be regulated by autocrine signaling, which needs further experimentation to be confirmed. Moreover, the astrocytic response to PDGF-BB involves secretion of CCL2 (131), which provides a context about how the BBB establishes a chemoattractant gradient to recruit peripheral immune cells.

Astrocytes secrete Shh to regulate BECs (130) and pericytes (132), but it seems that the expression of the Shh effector netrin-1 is suppressed by inflammation in BECs. In response to PDGF-BB, pericytes express many growth factors, including BDNF, βNGF, and VEGF (131), but ADAM10 cleaves PDGFRβ from the cellular membrane in the proinflammatory microenvironment (111). Homozygous loss of the PDGF-BB retention motif causes a deficiency in cerebrovascular pericyte coverage and a vast influx of immune cells into the brain (42), which suggests that PDGF-BB participates in BBB integrity maintenance.

Pericytes in the NVU play a central role in BBB maintenance, and their interaction with inflammation has not been completely elucidated. It has been suggested that pericytes can secrete many growth factors and cytokines in response to the PDGF-BB/PDGFRβ signaling pathway with the potential to regulate NF&ND (132). The detailed cellular mechanism of pericytes in response to neighboring cells has been reviewed by Sweeney MD (41), yet its roles in SCI need to be investigated further. In 2018, Duan et al. reported that PDGFRβ mural cells could excite glutamatergic neurons by releasing CCL2 in response to acute systemic inflammation. A recent investigation suggested that IL-1β contributes to the loss of retinal vascular pericytes as the key diabetic retinopathy, and a similar mechanism might possibly occur in the CNS.

### 4.3 NVU Uncoupling in Chronic Inflammation

NVU uncoupling is a synthetic effect that combines various factors, including protective and damaging, genetic, and environmental factors. Here, we mainly focused on functional loss caused by cytokines and immune cells in chronic inflammation. GM and its metabolites, such as ammonia (133), hydrogen sulfate (133), and trimethylamine-N-oxide (TMAO) (134), have a direct negative effect on BBB integrity. Considering their own specificities, it needs an encyclopedia to describe the related properties. Nevertheless, as xenobiotics, most GM and their metabolites stimulate BBB inflammatory responses.

It has been demonstrated that IL-1β can be transported from one side to another side of BECs *in vitro* and *in vivo* (33, 135), while brain injury increases their frequency transfer across the BBB (135). Furthermore, CXCL5/8 elevation has been found in the serum of MS patients; the interplay between serum CXCL5/8 and its BEC receptor CXCR2 can give rise to abnormal paraendothelial passage *via* Akt/protein kinase B by regulating the cytoskeleton and ZO-1 (118). Additionally, to mimic the proinflammatory profile of chronic diseases, three cytokines (IL-17, IL-6, and TNF-α) were grouped together to influence the *in vitro* model of the BBB, whose permeability increased significantly, whereas efflux transporters, such as MRP-1 and Pgp, were also upregulated (136).

Chemokines and cytokines not only bring about BBB hyperpermeability or disruption but also prime BECs for leukocyte trafficking. Among the various cytokines secreted by Th1 and Th17 cells, IFN-γ significantly increases the membrane expression of transmigratory members, such as ICAM-1, VCAM-1, and mucosal addressin cell-adhesion molecule (MAdCAM) 1, on BECs (**Figure 2B**). Moreover, together with the enhanced expression of molecules in the transmigratory pathway, IFN-γ could also regulate their relocalization in synergy with CD4+ TC translocation from the blood to the CNS; migration depends on the CCL21/CCR7 chemotactic pathway to activate downstream STAT1 (107). In turn, leukocyte transmigration would cause more severe damage to BBB architectures, including pericytes and BM. Meanwhile, the forced expression of Wnt/β-catenin signaling in CNS vessels restores BBB integrity and restrains the transmigration of CD4+ TCs and endothelial transcytosis (137).

Contrary to BECs, astrocyte responses to proinflammatory factors such as IL-1β and IFN-γ reinforce TJs by enhancing the expression of CLDN-1/4 and JAM-A at astrocytic endfeet (30), which suggests that the glia limitans is an immunoregulatory barrier that limits leukocyte trafficking. However, another question is also proposed: how do peripheral immune cells penetrate the abluminal side of the brain capillaries? Recently, it was reported that sustained peripheral inflammation activates brain microglia to transform into a phagocytic phenotype and internalize endfeet, which results in glia limitans breakdown (87). Although there is no report about how chemokines migrate through the glia limitans, it is known that astrocytes themselves secrete chemokines in response to cytokines, such as CCL2. In summary, a module is provided about the glia limitans disruption related to neuroinflammation, whereas the important questions remain to be investigated.

Pericytes interact with neighboring cells to regulate neurovascular functions; see reviews (41, 138). In the presence of chronic inflammation, brain pericyte coverage becomes sparse (46); recent progress suggested that pericytes could inhibit the expression of LAMs on ECs; loss of pericytes would increase LAMs expression together with leukocyte translocation into the brain (42). Moreover, TNFα or IL-1β can suppress the expression of α1-integrin, α-SMA, PDGF-Rβ, and CX-43 *in vitro* (46); subsequently, cytokines upregulate pericyte expression of C3, chemokines, and interleukins. Critically, chronic inflammation debilitated PDGF-BB/PDGFR signaling and precipitated BBB leakage, increasing the risk for amyloid β (Aβ) and neurofibrillary tangle accumulation (41). Additionally, secreted chemokines induce leukocyte extravasation, and pericytes guide interstitial leukocyte migration by physical contact to enhance immunosurveillance (139).

The aging brain is an important research field that requires long-term effort, and the examination of the crucial role of BBB “leakage” in NF&ND is a major progress in neuroscience and physiological aging. Increasing age is concomitant with increasing brain microvessel permeability, basal lamina thickness, and the extended size of astrocyte endfeet, yet the function of P-glycoprotein decreases (140). BBB changes in the process of aging might not be similar between humans and rodents or primates; see reviews (141). Briefly, the aging capillary wall became thick in humans but not in rats and monkeys, but the decrease in the BEC number and tight junction protein was shared by the three species (141). In the aging brain, the number and size of astrocytes increase together with their GFAP, while pericytes lose their special ultrastructure and become migratory (141).

### 4.4 Morphogen Signaling of NVU interrupted by Chronic Inflammation

NVU coupling is a matter concerning of BBB integrity (21) and brain homeostasis. BBB formation and maintenance rely heavily on the canonical Wnt/β-catenin, Sonic Hedgehog, PDGF-β, and TGF-β signaling pathways (21, 142). In contrast, cytokines, reactive oxygen species, and some other inflammatory mediators likely cause aberrant BBB permeability or disruption (13). Additionally, inflammatory factors from both sides of the BBB stimulate proinflammatory signaling pathways (Jak-STAT, NF-kb, and NLRs) in BECs, pericytes, and astrocytes and potentially precipitate the degeneration of the BBB. Therefore, we assume that the maintenance cues of the BBB are disturbed in the presence of SCI and/or neuroinflammation and mainly focus on how proinflammatory cytokines disrupt the morphogen signaling (Wnt/β-catenin and SHH) that lead to NVU breakage.

#### 4.4.1 SCI Disrupts Wnt/β-Catenin Signaling

The study of canonical Wnt pathways in the genesis and maintenance of the BBB is continuously moving on. Wnt7a/b-Frizzled receptors and Norrin-Frizzled receptors are the two major Wnt family members in the CNS (20, 143). Members of Wnt signaling remain transcriptionally active in adult BECs, albeit their vasculature expression is very low (144). Moreover, canonical Wnt signaling is concomitant with the expression of Cldn-5 and Glut1 and negatively associated with plasmalemma vesicle-associated protein (Plvap) (20). GPR124 is an orphan G-protein-coupled receptor (142), while the crosstalk between Wnt signaling and Gpr124 could strengthen BBB functionality (144). Recent findings suggested that polymerization of Wnt/Frizzled/Lrp5/6 signalosomes not only requires Dishevelled to recruit Gpr124 but also requires Reck to bind Wnt7 (145). Reck is a special protein with an N-terminus CC4 domain responsible for GPR124 binding and WNT7A/WNT7B recognition (146). Reck and Gpr124 transduce Wnt7a/Wnt7b signaling into BECs to regulate the BBB (147).

In 2007, Duan et al. found that beta-catenin is a negative regulator of the inflammatory NF-kappaB signaling pathway (148). To date, some associated mechanisms have been clarified. For example, Wnt/β-catenin signaling in CNS vessels could limit the transmigration of immune cells into the brain parenchyma and partially restore functional BBB integrity in EAE/MS models (137). Targeted inhibition of the Wnt/β-catenin pathway in the CNS endothelium accelerated the endothelial transcytosis of CD4(+) T cells into the CNS as well as increased VCAM-1 and Caviolin-1 expression (137). Recently, Jridi et al. analyzed the role and function of the Wnt pathway in inflammatory diseases (149). Nevertheless, how inflammation and Wnt signaling interact in the NVU is still unknown.

Thus far, it is known that astrocytes are the major source of Wnt signals in NUV; when Frizzled Receptor of BECs binds to Wnt, GSK-3β will be sequestered from its substrate β-CATENIN, a change advantageous for β-CATENIN to keep away from destruction complex and to ensure its stability for downstream effects (150). Nevertheless, the balance could be broken via GSK-3β activation by proinflammatory cytokines and metabolic status. GSK-3β is likely one of the hub molecules that mediates the crosstalk between Wnt signaling and systemic chronic inflammation, but its role in inflammation requires more comprehensive investigation (151). Moreover, sporadic investigation suggested the importance of Wnt signaling in BBB integrity and brain homeostasis. For example, p-glycoprotein (p-gp) and some other multidrug efflux transporters were upregulated by inhibition of GSK-3 or activation of Wnt/β-Catenin (152). In the aging hippocampus, age-increased inflammation (including TNF-α and NF-кB signaling) was accompanied by AKT/GSK-3β activity, while the decrease in dishevelled 2 (DVL2) expression might determine the strength of canonical WNT/β-CATENIN signaling (153). Interestingly, a few cell structural proteins have displayed opposite regulatory trends in response to Wnt signaling and inflammation, such as CLDN5, VCAM-1, and Caviolin-1, and the underlying mechanisms need further investigation.

#### 4.4.2 SCI Interrupts SHH

Similarly, astrocytes are the major source of sonic hedgehog and act to maintain BBB integrity and CNS immune privilege in the adult brain (26). The Shh pathway plays a protective role after injury to the CNS by limiting neuroinflammation (154, 155). *In vivo*, deficiency of Shh signaling exacerbates neuroinflammation across the brain, brainstem, and cerebellum (155). Both endogenous and exogenous Shh signaling have the potential to mitigate inflammatory immune cell profiles and restrain the release of proinflammatory cytokines (154, 155) with better pathological outcomes (154). The role of SHH in angiogenesis and maintenance has been reviewed by Chapouly and his colleagues (156), and SHH and Wnt signaling could interact with each other to regulate the BBB, which was reviewed by Gozal and his colleagues (157).

SHH released from astrocytes was suppressed by interleukin-1β (IL-1β), which could be derived from activated microglia or peripheral circulation and could abolish Shh protection of BBB integrity (158). It is possible that IL-1β turns astrocytes to produce proinflammatory factors, such as CCL2, to promote BBB disruption and neuroinflammation. Because TJ expression is positively associated with SHH signaling, astrocytic SHH-directed BBB restoration would provide a promising therapeutic perspective (158). Interestingly, endothelin-1 (ET-1) is an identified molecule that downregulates the Shh signal to uncouple the NVU, which is the downstream effector of the IL-6/JAK/STAT3 signaling pathway (159). Hence, it is reasonable to infer that circulating IL-6 would enhance ET-1 activity and interfere with the SHH signaling pathway in BECs (160). Vice versa, Shh could interact with NF-κB p65, and enhanced Shh expression rescued oxidized low-density lipoprotein-induced endothelial apoptosis by suppressing the NF-κB pathway (161). Conversely, increased NF-κB signaling could inhibit the Shh pathway (162). It is well known that the NF-κB pathway is downstream of proinflammatory cytokines, TLRs, and TNF-alpha receptors.

## 5 From GM-SCI to NF&ND

### 5.1 GM and Gut Mucosal Immunity

Approximately 10^13^–10^14^ living microorganisms reside in the human digestive tract with the capability to provoke mucosal immunity. In the long duration of coevolution, the interaction between the commensal GM and host immunity reaches a balance of symbiosis. The mutualism between humans and their intestinal commensals produces many benefits, including vitamin synthesis and nutrient absorption. Because the gut tract connects the outside environment, the balance is liable to go awry and become pathogenic (1). However, host immunity places selection pressure on GM composition to hold extreme pathogenic changes. For example, in age-associated MetS, TCs regulate the balance between *Desulfovibrio* and Clostridia to prevent obesity, and the functional loss of TCs causes improper IgA attack on Clostridia (163). When the compensatory mechanisms begin to lose their efficiency in correcting the deviation, gut inflammation and autoimmunity would be primed for expansion and spread; see reviews (164, 165). Generally, gut microbial dysbiosis is often associated with chronic diseases, such as autoimmune diseases and diabetes, whose initiation and progression are latent and imperceptible. Undoubtedly, diet and age are the major covariates in GM composition changes (section 5.2) (166, 167) (**Figure 1A**).

IB maintains the balance between nutrition absorption and pathogen prevention in the mammalian gut, which is composed of a physical layer mainly composed of epithelial cells interconnected by TJs, covered by a mucus layer with antimicrobial peptides and secreted IgAs on the luminal side, and immunological layers, including immune cells in the lamina propria (LP); see reviews (168, 169). M cells, microfold cells, are specialized epithelial cells covering the dome of Peyer’s patches (PPs) and facilitating immune surveillance, where intestinal pathogens or antigens are ingested and transported into lymphoid follicles (170) (**Figure 2A**). Considering gut mucosal immunity, T-cell subsets play a protective role in maintaining IB homeostasis by aging gut commensals (164, 169, 171), but the adverse aspects of TCs are the drastic immune response causing IB “leakage” (**Figure 1B**).

Here, we summarize several points of immune responses by the interaction of gut mucosal TCs and luminal commensals as the origin of inflammation. First, intraepithelial T cells (IETs) are tissue-resident adaptive immune cells that can quickly respond to antigen challenges independent of peripheral T-cell recruitment, thus contributing to the persisting low-grade inflammation of chronic gastrointestinal diseases (172). Second, CD8+ tissue-resident memory (TRM) TCs mainly reside in intraepithelial containments where they can recognize the specific antigen as sentinels; CD4+ TRM cells mainly reside in the LP in healthy conditions, whose activation mainly relies on antigen-presenting cells and move up from the LP into epithelial cells in disease (173). Third, serum amyloid A (SAA) proteins are the by-product of intestinal epithelial cells (IECs) in inflammation, which could induce the production of pathogenic Th17 cells differentiated from naïve CD4(+) TCs (174). Fourth, Tregs suppress inflammatory cells to maintain quiescent mucosal immune responses (175). Nonetheless, it should be recognized that the translocation of luminal antigens into the internal environment is a reason to arouse stronger systemic inflammation, but the interaction between T lymphocytes and their surroundings is one of the determinants to block the spread of inflammation; for more information, see reviews (164, 169, 171, 175, 176).

On the other hand, gut lymphocytes in the digestive tract are in a quick homing and emigration equilibrium with extensive effects on immunoregulation of gastrointestinal tract and beyond. In lamb ileal PP, the number of B cells is much higher than that of TCs, and both of them emigrate quickly (177). The B-cell emigrants of ileal PP contribute 8.9% and 6.8% to blood and spleen B-cell pools, respectively, while T emigrants account for 1.5% of the various tissue T-cell pools (177). By molecular recognition, intestinal homing T lymphocytes express the α4β7 or α4β1 integrin to be identified by VCAM-1 on the surface of endothelial surface of the gut postcapillary venules; chemotaxis also contributes T lymphocyte homing and accumulation in the bowel for its expression of CCR9 with the potential to react with its ligand CCL25 secreted from the inflammatory colon (171). Moreover, memory B cells (BCs) adopt a similar mechanism for their intestinal homing by recognizing VCAM-1 with alpha4beta7+ or alpha4beta7- integrin on their plasma membrane (178). Generally, the lymphocyte homing mechanism enhances the capability of gut mucosal immunity to clear invaded commensal microbes and provides the opportunity for immunosurveillance in distal organs.

### 5.2 GM and IB Breakage

Gut mucosal surfaces are the first-line barrier to dialog with the GM, whose permeability or disruption is susceptible to damage from both pathogens and physical trauma. For deregulation, the integrity and continuity of the gut epithelium could be disrupted by enterocolitis, and at least three possible mechanisms for leaky gut module have been addressed, namely, disintegration of the junctional complexes induced by TNF, pore pathway regulated by IL-13, and epithelium loss caused by cellular apoptosis (179). To engage with mercurial lumen environments, IECs contain an inflammasome apparatus ready for apoptosis; caspase-8 launches extrinsic apoptosis as a molecular switch to prevent tissue damage (179).

IB is abundant for unconventional lymphocytes such as innate lymphoid cells (ILCs), γδ TCs, and mucosal-associated invariant T cells (MAIT) with the capability to repair mucosal damage. It has been recognized that dysregulation of the immune response facilitates gut mucosal inflammation. Thus, MR1−/− NOD mice are deficient in MAIT development in the thymus and cannot prevent FITC-dextran penetration from the gut into the blood. Indeed, many investigations have provided the functional roles of immune cell dysregulation in IB deficiency per se. Therefore, it is imperative to setup the holistic views about how GM dysbiosis triggers mucosal immune dysregulation to injure gut epithelium permeability initially. It has been suggested that gut mucosal homeostasis is maintained by γδ TCs that can be divided into two functionally distinct subsets, namely, the “γδT-17” and “γδT-IFNγ” subsets (180). The γδT-IFNγ subset secretes keratinocyte growth factor (KGF) to maintain IB integrity. Nevertheless, secretion of IFN-γ is a potential damaging factor to the intestinal epithelium (181). However, it is still unknown how the GM and its metabolites impair the balance with gut immunity and mount inflammation at the gut epithelium layer. Under actual conditions, the mucus layer attenuates the exposure of gut epithelial cells to unfriendly lumen contents and mitigates the occurrence of inflammation. Goblet cells are the major player contributing to mucus layer formation, but how the gut microbiota regulates MUC gene expression and goblet cell regeneration *via* gut mucosal immunity needs more extensive explorations; see reviews (182, 183).

Even now, leaky gut remains an assumption or overreaching conclusion due to the lack of persuasive clinical evidence. In 2020, D’Amato and his colleagues reported that young adult mice displayed impaired spatial learning and memory after they received the transplanted microbiota from age-matched donor mice, while they did not present with abnormal gut permeability or increased cytokine levels (184). This is an important research worthy of further extensive effort to understand the underlying puzzles. For example, are there some strains from elder gut microbiota with the capability to produce small neurotoxic molecules? Clinically, systemic reviews strongly support IB changes under chronic disease conditions. A meta-analysis with 14 eligible case–control studies found that nonalcoholic fatty liver (NAFL) patients had a significant penetrance of intestinal permeability (IP) over healthy controls and severity, defined by nonalcoholic steatohepatitis (NASH) (185). In the event of aging, IB is subject to extreme changes, such as mucus layer thinning, interepithelial gap expansion, and attenuation of TJ components. Another clinical trial suggested that the serum zonulin, a surrogate biomarker of aging-associated leaky gut, was higher in elders than in young adults, with a strong implication to support the coexistence of the leaky gut and frailty (186). Animal experiments on baboons suggested that age-related intestinal permeability increase was positively correlated with inflammatory cytokines and was negatively correlated with junctional complexes by colonic biopsy (187). Collectively, the “leaky” gut is becoming the irresistible supposition that underpins the pathophysiology of many chronic diseases because unresolved gut mucosal inflammation and penetrating xenobiotics can be transmitted to distal tissue or organs (188).

### 5.3 Gut Inflammation Spreading Routes

Two major conduits for the spreading of gut inflammation are suggested: the lymphatic drainage pathway and systemic circulation after the disruption of gut vascular barriers (GVBs). Lymphatic drainage ensures the unique property for immunity to be both local and systemic (**Figure 2**). That is, the immune response started at one site and could be reactivated at distant tissues or organs. The intestine is one of the major sites for immune cell priming for the immune response both within and outside of the digestive tract (171). Peyer’s patches, the intestinal epithelium, and the LP reside in different lymphocyte populations. Primed T and B lymphocytes migrate out of the PP, join the lymphatic fluid, flow through the mesenteric lymph nodes, and enter the thoracic duct and blood circulation, including CD4+ TCs (171) and BCs (189). Several articles have investigated the emigration of gut-derived lymphocytes in pigs and sheep and have demonstrated that only a small portion of recirculating lymphocytes home back to the intestine. For example, BrdU-labeled TCs (10%), BrdU-labeled B cells (2%), and BrdU-labeled γδ TCs (22%) reappeared in intestinal lymph (190). Furthermore, gut-derived γδ TCs render the major counterparts in the peripheral counterpart (190), and some of them grow with proliferation capacity (190). In 2020, Pasciuto et al. reported an example of how peripheral CD4+ TCs migrate into the brain parenchyma and educate nascent microglia to maturation under healthy conditions (81). Strikingly, CD4 TCs in the brain after experimental mice showed a higher number of CD4 TCs after a 6-week cohousing period than GF and SPF mice (81). It is assumed that the cohousing procedure increases the microbial exposure to the experimental mice. As for how gut-educated lymphocytes interact with the CNS, more studies are needed to illustrate the comprehensive mechanisms.

The functional architecture of GVB, the gut vascular unit (GVU), shares a certain similarity with the well-characterized BBB; see reviews (191). Briefly, endothelial cells of GVU interact with pericytes and enteric glial cells and form junctional complexes to enclose the lumen of blood vessels. Noticeably, enteric glia release S-nitrosoglutathione to strengthen TJs to maintain paracytosis. Similar to the NVU, canonical Wnt/β-catenin is involved in GVU maintenance. Although both GVU and NVU maintain the balance between nutrition absorption and blocking detrimental substance translocation, GVU allows larger molecule diffusion up to 4 kDa. GVB is the last line of defense against luminal pathogens entering the blood because the disruption of GVB occurs after the inefficient clearance of inflammatory mediators by gut mucosal immunity. Under gastrointestinal diseases, bacteria and their debris or metabolites may translocate into the systemic circulation and spread to distal tissues and organs (192). The deepest defense layer of intestinal GVB is spoiled by entering pathogens and promotes systemic inflammation and spread from the liver to the brain, a route called the gut–liver–brain axis (191).

### 5.4 Systemic Inflammation Induced by Gut Microbiota

The interplay between the GM and gut immunity determines the incidence and spread of inflammation. Although gut commensals can be either beneficial or detrimental, they become detrimental when IB is impaired (“leaky gut”) (**Figure 1B**). Once microbial debris and its metabolites translocate to subepithelial containment, the initiated immune responses become stronger and spread *via* systemic circulation (193). Meanwhile, the pathogenesis of systemic inflammation associated with GM would be miscellaneous, but several very promising common factors would favor the commencement and progress of chronic systemic inflammation.

First, a crucial change caused by gut commensal dysbiosis is IB disruption, whose underlying mechanisms depend on the balance of gut mucosal immunity and luminal microorganisms. Since birth, the gut microbiota participates in IB establishment and maintenance *via* interaction with the host immune system; conversely, host gut immune maturation relies on the coevolved specific microbiota (194). As age increases, gut dysbiosis changes the inflammatory status of the gut mucosa. The concept of host-specific microbiota suggests that the host has the capability to keep colonized gut microorganisms relatively stable, and the oscillation of the microbial ecosystem alternates gut mucosal immunity to maintain IB integrity and host health. Gut dysbiosis includes the compositional changes of the gut microbial ecosystem with the possibility of modifying mucosal immunity. Regardless of how they evolved, the gut microbiota remain the external stimulus for innate and adaptive immunity (see reviews) (164, 169, 171, 176). Under healthy conditions, a low level of baseline inflammation is persistent in the IB, but GM dysbiosis often disrupts homeostasis and intensifies inflammation in the IB. Of note, gut mucosal immunity cannot always clear the pathogens derived from the dysbiosis ecosystem. Hence, sustained dysbiosis leads to gut immune dysregulation and unresolved local and/or systemic inflammation. Gut dysbiosis could have been induced by gut viruses that form symbiosis with some commensals and are detrimental to other microbiota; however, the occurrence of this phenomenon has not been discovered in the gut (**Figure 2B**). If that were the case, the homeostasis of gut mucosal immunity would be changed to a different status and precipitate the origins of SCI (195).

Second, the detriment of the gut virome is more than changing GM composition; it is also a hidden risk factor that could not be negligible, see reviews (196–198). Bacteriophages are not passive members of the gut and are interweaved with bacterial and host immunity as a trio (196). Assumedly, gut viral particles would be released into the lumen under appropriate conditions, and the released nanoparticles would probably penetrate through the gut epithelium to stimulate stronger immune responses. Whether mucosal immunity would develop an efficient strategy to eliminate bacteriophages needs further extensive mining. Strained by the related knowledge and techniques, an early longitudinal gut virome study suggested intraindividual stability and high interindividual diversity; however, with the popularization of advanced sequencing techniques, a large amount of gut “dark materials” of bacteriophages have been identified (198). Tropism of bacteriophages influences the community structures of commensals. For example, stable GM communities with crAss-like and Microviridae bacteriophages coexist abundantly with the bacterial genera *Bacteroides*, *Prevotella*, and *Faecalibacterium* (197). An intriguing finding is that the increase in the temperate phage number is a specific change in Crohn’s disease, a distinct finding contrary to the previous conclusion of no viral changes in IBD (199). Furthermore, bacteriophages were associated with IB permeability. Under experimental conditions, the administration of the bacteriophage cocktail caused less motility, mess hair and weight loss, and the level of circulating immune complexes (CIC) increased 2.5-fold on the 10th day (200). Another recent greatest progress is the update of the Metagenomic Gut Virus catalog, composed of 54,118 candidate viral species sampled from 11,810 individuals; more than 90% of species had not been found before (201), which would accelerate hypothesis-driven studies in clinical and aging. In 2020, Geogory et al. described the human gut virome pattern with increasing age, which indicates an unexpected fact that overall viral richness decreases significantly from adults to elderly individuals in both Western and Chinese cohorts (202). In sum, the potential role of gut bacteriophages in health, diseases, and aging has just started to be realized, and future investigation would bring surprising achievements (see reviews) (203).

Third, gut dysbiosis is also subject to dietary habitats or becomes severe with age (section 6) (**Figure 1A**). In 2008, Cani et al. reported that high-fat diet-induced GM dysbiosis caused metabolic endotoxemia and serum inflammation (166); later, dietary-associated metabolic endotoxemia was suggested as a risk factor for several chronic inflammatory conditions, including obesity and diabetes (204). Paneth cells are one of the gut epithelial members with the ability to secrete lysozyme and defensins by sensing luminal commensal and antimicrobial peptides *via* the TLR signaling pathway. Lately, it was found that a Western diet (WD) would vitiate the presence of Paneth cells in the gut (205), while the WD is noted for its high fat and high sugar as the obesogenic diet. However, the reduction in Paneth cells was not reproduced in germ-free mice fed a WD but was induced after the gavage of *Clostridium scindens*, a strain with the ability to convert primary bile acids (205). Moreover, a high-fat and high-sugar diet could reduce the number of gut intraepithelial T lymphocytes (IELs) significantly accompanied by metabolic hyperactivity if IELs could not be replenished (206); further research indicated that metabolic suppression of IELs is mediated by GLP-1 expression as a gatekeeper to regulate enteroendocrine activity (206). Together, gut mucosal immunity, gut microecosystem imbalance, and modulating factors not only determine regional inflammation but also influence the body systemically.

Fourth, metabolites from the GM have profound effects on IB and systemic inflammation, including small molecules and microbial components (207); short-chain fatty acids (193) (**Figure 1H**), bile acids (193), nitrogen oxide (208), methane (209), and hydrogen gas (209) can exert anti-inflammatory effects. LPS might amass in the brain vasculature and work as a cofactor to aggravate ischemic brain damage, a report suggested in 2020 (210), but their direct roles in brain function need further investigation. In addition to LPS, other gut-derived proinflammatory factors, including TMAO (193) and ammonia (211), have been reported (**Figure 1G**). However, a comprehensive understanding of gut-derived metabolites remains difficult. Sulfidogenic bacteria in the human gut produce hydrogen sulfide; the role of hydrogen sulfide could not only promote NF occurrence but also protect the luminal surface of the colon (133, 212). Phenol and indole derivatives in the serum of MS patients were gut-microbiota-generated metabolites, including p-cresol-sulfate, indoxyl-sulfate, and N-phenyl-acetyl-glutamine, which had chronic neurotoxicity to the cultured neurons (213). *In vivo*, these bacterial origins were negatively correlated with the patient’s cortical volume and positively correlated with neurofilament light chain (213). Altogether, the gut microbiota not only induces systemic inflammation but also releases neurotoxic metabolites to promote MS.

### 5.5 GM-SCI Contribution on NF&ND

Both preclinical and clinical findings strongly suggested that GM and its metabolites influence the onset and progression of NF&ND (11, 214, 215) (**Figure 1**). Without significant elevation of proinflammatory factors, an aging-like phenotype of microglia appeared in the hippocampus of young adult mice receiving gut microbiota from aged mice by FMT; it is noticeable that the serum proinflammatory factors of young adult mice were not elevated significantly (184). Actually, the incidence and progress of NF&ND-associated intestinal microorganisms is a long-term, multifactor, and multistep process, a time span calculated in years or decades at least, from gut commensal dysbiosis to the CNS pathology that is not only affected by host genomics, lifestyle, age, sex, comorbidity, and polypharmacy but also accompanied by tissue damage, regeneration, remodeling, and gradual senescence resulting from random accumulated molecular errors, sustained low-grade inflammation, and immune dysregulation (18).

For example, a cross-sectional study in 1996 found increased intestinal permeability and CD45RO CD20+ B cell levels in MS patients (216). Later, it was found that active brain autoimmunity was associated with excessive gut Th17 cell expansion in MS, and the production of Th17 cells was negatively associated with Prevotella strains (217). Recently, Cox and his colleagues demonstrated that *Akkermansia* from MS patients could repress RORγt+ and IL-17-producing γδ TCs, whose presence is reversely correlated with disability (17). A successful MS mouse model was induced by exposure to a peptide (VP2121–130) derived from TMEV that could induce BBB disruption and CD8+ TC infiltration of the mouse CNS (110). TMEV belongs to the Picornaviridae family, and its presence can be found in feces from naturally infected mice. For another example, the microbial taxa alterations in PD patients are quite inconsistent, and lifestyles, including coffee consumption and constipation, are important covariates influencing microbial composition; interestingly, *Faecalibacterium* and *Ruminococcus* in PD patients were most steadily diminished in PD fecal samples (218). Interestingly, c2-like and 936-like lactococcal phages from dairy may cause PD in humans (200), although how they interact with the host immune system is unclear. For recent progress of GM in AD pathophysiology *via* immune dysregulation, see review (219). Although association studies suggest a connection between NF&ND, it is still not known how the GM interacts with extraintestinal tissues mechanistically.

Notably, the advancing knowledge of the microbiome and its metabolites (29, 220), PRRs (125), host inflammasomes (221), CD4+ TCs (171, 222), CD8+ TCs (110, 169), Th17 cells (72, 174), Tregs (223, 224), and γδ TCs (180, 225) provides crucial contexts to scrutinize and decode the intricate relations between GM and NF&ND, especially to determine how circulating inflammatory mediators and immune cells cause the “leakage” of the brain vasculature. Although the mucosa of the digestive tract is the main interface for the strong immune challenges to the resident microbiota, its role in brain health is supported by other protective mechanisms in the body in early life. For example, the reticuloendothelial system (RES), including the liver and spleen, eliminates translocated endotoxins and bacteria to maintain internal homeostasis (226, 227). Following ingestion of the pathogen debris, RES could secrete inflammatory mediators into the blood circulation and change the inflammation status. Consequently, the restricted low inflammation at the gut epithelium ceaselessly occurs, but it is difficult for gut-derived pathogens to attack the CNS and induce NF&ND before the functional deficits of RES in most times of life. Nevertheless, the elevated cytokines and the activated lymphocytes impose the pressure of tissue remodeling and abnormal transcription programs on the NVU before clinical symptoms arise.

To date, both cellular and humoral immunity prompted in gut-associated environments have been suggested to have a strong association in NF&ND. In contrast to other CD4+ Th1 cells, peripheral circulating Th17 cells actively transmigrate across the BBB (62). Usually, germ-free mice lack Th17 cells, whose number is mainly dependent on microbiota colonized in the gut of GF mice (174). Moreover, pathogenic Th17 cells could be induced by serum amyloid A (SAA) proteins *via* the MAPK pathway instead of SMAD2/3, similar to TGF-β. As a result, SAA-induced Th17 cells demonstrated a “pathogenic” Th17 signature with expression of IL-23R (174). Nevertheless, SAA can be produced in multiple sites, including the intestine and liver. Alternatively, it has been demonstrated that IL-17+ γδ TCs are neurotoxic, and naïve γδ TCs are irresponsive to TLR ligands; however, the factors released by activated microglia *via* TLR2/4/9 signaling stimulate naïve γδ TCs to express IL-17 with neurotoxic capacity (228), which recapitulates one of the possible NF&ND mechanisms. However, IgA-producing plasma cells (IgA+ PCs) or B cells are exceptional because their secreted antibody is specific to gut-derived antigens (96, 229, 230). It has been found that gut-derived IgA+ PCs release IL-10 to suppress NF (96) and extravasate into the meningeal region to defend against fungal infiltration (229). Furthermore, IgA+ PCs found in the inflamed CNS were gut MS-associated taxa, providing pervasive evidence of distant effects on gut mucosal immunity (230). In summary, the incidence of NF&ND is most likely caused by GM *via* chronic systemic inflammation, but there remain many unknown gaps from gut microbiota to pathologies of NF&ND, and the complete comprehension of GM-SCI-induced NF&ND would improve the medical strategies of NF&ND intervention.

## 6 GM Effect on Aging and Brain Aging

Human commensal homeostasis determines host aging, which is supported by the finding that germ-free mice are more likely to live longer than 600 days and less likely to suffer from age-associated inflammation and macrophage dysfunction (231). Furthermore, the disturbance of GM composition in elderly individuals is assumed to be one of the major sources contributing to inflammaging. This assumption, supported by the age-associated “leaky” gut, translocation of GM-derived pathogens into circulation, elevated TNF, and senescent macrophages with enhanced IL-6 secretion, has been demonstrated in animal models (231). For more information about visceral inflammation and CNS stress, see reviews (232). To be a part of human commensals, it is still difficult to evaluate the GM contribution in aging and brain aging independently.

On the other hand, far from being demonstrated as the triggering factor, GM and its products would be an indispensable part of the aging process. First, research on GM and human aging found the compositional difference between biological age and chronological age. Biological age is indicated by the physical frailty index, which is negatively correlated with GM diversity (233), whereas advancing chronological age displayed a trend of increased GM diversity and variability (233). Compared to early life, the shrinkage of GM variability is reversed to biological age, implicating the role of gut dysbiosis in somatic decline. Second, together with higher LPS in plasma and feces, the Firmicutes-to-Bacteroidetes ratio increases with age. Moreover, inflammation and cellular senescent pathway members, NF-κB and p16, respectively, were elevated in the mouse colon; the activation of NF-κB in response to the LPS fraction of fecal lysates is dependent on TLR4, while p16 could be activated by other unknown mechanisms in the deficiency of TLR4 (234). Third, transplantation of GM from aged mice stimulated intestinal inflammation in GF mice, accompanied by the influx of pathogenic contents into the systemic circulation and the activation of T lymphocytes; the analysis of metagenomics suggested that the pathogenesis may result from the changed ratio between *Akkermansia* and TM7 bacteria and Proteobacteria in aged mice (235). Until now, the evidence that physical aging is associated with GM components has been demonstrated, but the causal relationship between inflammaging and altered gut microbiota is unclear.

To find out the optimized underlying mechanisms, centenarians and typical neurodegenerative diseases are chosen to break through the perplexing problems. Centenarians are a healthy aging paradigm with less susceptibility to chronic inflammation. One investigation found that centenarians were rich in a distinct GM with the ability to produce special secondary bile acids (BAs); among these BAs, isoalloLCA not only is a newly identified BA member but also has strong antimicrobial activity (236). The strains for Odoribacteraceae isolated from centenarian feces were efficient in isoalloLCA production, which would decrease the pathobiont load (236). Colonic RORγ+ FOXP3+ Treg cells are upregulated by the secondary BA pool by BA nuclear receptors and further alleviate inflammatory susceptibility and maintain colon immune homeostasis (237). Hence, GM from the centenarian provides an inflammation-suppressing environment, which sounds most reasonable and provides a certain context for scrutinizing detrimental factors in biological age. In another fecal transplantation from the elderly to study AD, *Paenalcaligenes hominis* was found to be associated with colonic inflammation and cognitive degeneration. An in-depth study suggested a new way for *P. hominis* to impair cognition through the vagus nerve in the form of extracellular vesicles (EVs) (238); LPS from *P. hominis* could also cause cognitive deficits, while celiac vagotomy had no effect on its pathogenicity (238).

GM-induced brain aging provides an indicator of overall physical senescence for the following reasons (18, 232, 233). Untreated “leaky gut” leads to the influx of luminal pathogens, causes various pathological changes along the spreading routes, and eventually precipitates the dysfunction and degeneration of the brain (232). In such scenarios, the brain is almost the last affected internal organ compared to the intestine, liver, spleen, lung, aorta, and heart. In fact, GM-associated comorbidities, including inflammatory bowel diseases (IBDs), ulcerative colitis, NAFL, atherosclerosis, type 2 diabetes, and MetS, have been diagnosed before the emergence of clinical neural diseases, iterating one of the essential contexts of chronic inflammation of the aging process from gradual functional loss of peripheral organs to frailty and brain aging. Although when and how the vagus pathway of *P. hominis* becomes apparent needs further examination, it is certain that the presence of *P. hominis* would worsen BBB corruption and NF&ND and accelerate aging (238).

Altogether, the gut is the major site for immune cell education, and the circulating immune cells in the peripheral blood of elderly individuals (ranging from 60 to 80 years old) have attracted the interest of geriatric studies, which would provide clues regarding brain aging (section 5.5). Importantly, effector and inflammatory phenotypes prevail as age increases, while TCs and BCs from older people decrease approximately 10% of the total PMBCs, especially naïve CD4 and naïve CD8 (239), which might imply compromised hemopoiesis for the accumulated temporal damage to self-renewal and increased microbial invasion owing to the attenuation of barrier defense (239). Understanding the influence of gut-educated immune cells on CNS pathogenesis has become perceptible [see reviews (18, 240, 241)], a topic possibly related to common mucosa immunity (242).

## 7 Conclusion and Perspectives

Knowledge about the GM, chronic inflammation, and NF&ND strongly suggests a sequential causality and consequence relationship, and sustained immune dysregulation and barrier breakage are the most crucial mechanisms underlying the initiation and progression of NF&ND. Although several approaches that have been developed to rescue GM dysbiosis and barrier dysfunction have been reported to be efficient, their long-term effects in disease prevention and healthy aging need further observation. The current mechanistic understanding of the GM and its pathogenesis is still not sufficient to provide or develop new and efficient treatments. The occurrence of NF&ND related to GM and its metabolites is a multifactor, multistage process based on genetic–environmental interactions. Modern techniques, including metagenomic, meta-transcriptomic, and metabolomic techniques, and recent combinations of single-cell and spatial transcriptomics would pave the way to clarify the pathogenesis.

Dysregulation of gut mucosal immunity contributes to the inflammatory profile of systemic inflammation. Gut mucosal immunity is stimulated constantly by the pathogenic gut symbiosis, repeated dietary antigens, and unhealthy lifestyles, which makes local and systemic inflammation unresolved. Because histological barriers defend against xenobiotic infiltration and maintain internal homeostasis for physiological function, theoretical extrapolation suggests that BBB dysfunction provides the premise for brain aging as well as preclinical animal experiments and clinical investigations (243). Moreover, because not all host repairment might strengthen the brain vasculature wall perfectly, the GM has begun to be considered a crucial player in stroke (rupture of brain vessels), a global destructive brain disorder.

In conclusion, the philosophical idea that the gut microbiota is associated with NF&ND and brain aging is increasingly supporting experimental evidence. Systemic chronic inflammation plays a central role in the progression of NF&ND. Mechanistically, IB disruption would make the situation more serious and initiate chronic systemic inflammation, which further modulates the NVU structure or uncoupling by signaling pathways. Even if it is not specific to the disease pathophysiology, chronic inflammation would cause gradual tissue, organ, and repairment degeneration and functional loss. For a more precise understanding, it is necessary to clarify the relationship of the gut ecosystem, gut mucosal immunity, molecular profile of SCI, and NF&ND together. Particularly, it is important to determine the inherent connection between the SCI profile and the pathophysiology of extraintestinal organs and, conversely, to investigate how the interplay between the gut ecosystem and gut mucosal immunity would illiciate the process of NF&ND to recapitulate SCI profiles in BBB breakage.

## Author Contributions

YM contributed to manuscript writing and editing. YD, LZ, JY, YL, XL, and GZ provided beneficial advice on the manuscript. SC and XH supported the completion of the manuscript. HX and BD guided the composition of the manuscript. All authors contributed to the article and approved the submitted version.

## Funding

This work was supported by the Provincial Agency for Science & Technology, Sichuan (Grant No. 2016JY0125), National Key Research and Development Program of China (Grant No. 2018YFC2000400).

## Conflict of Interest

The authors declare that the research was conducted in the absence of any commercial or financial relationships that could be construed as a potential conflict of interest.

## Publisher’s Note

All claims expressed in this article are solely those of the authors and do not necessarily represent those of their affiliated organizations, or those of the publisher, the editors and the reviewers. Any product that may be evaluated in this article, or claim that may be made by its manufacturer, is not guaranteed or endorsed by the publisher.

## Glossary

AJs: adherens junctions
ALS: amyotrophic lateral sclerosis
AQP4: aquaporin 4
BM: basement membrane
BAs: bile acids
BBB: blood–brain barrier
CSF: blood–cerebrospinal fluid
BECs: brain endothelial cells
CCR4: C-C motif chemokine receptor 4
CNS: central nervous system
Ccl6: chemokine (C-C motif) ligand 6
CLDN: claudin
Cx43: Connexin 43
CRP: C-reactive protein
CXCL1: C-X-C motif chemokine ligand 1
CXCR2: C-X-C motif chemokine receptor 2
DCLNs: deep cervical lymph nodes
DHH: deserted hedgehog
ECs: endothelial cells
ET-1: endothelin-1
FGF: fibroblast growth factor
GF: germ free
GSK-3β: glycogen synthase kinase 3beta
GM: gut microbiota
GM-SCI: gut-microbiota induced systemic chronic inflammation
ILCs: innate lymphoid cells
IL-1β: Interleukin-1β
IL-6: Interleukin-6
IB: intestinal barrier
IECs: intestinal epithelial cells
IETs: intraepithelial T cells
LAMs: low leukocyte adhesion molecules
LP: lamina propria
LPS: lipopolysaccharide
MCP-1: macrophage cationic peptide 1
MRI: magnetic resonance imaging
MMP: matrix metallopeptidase
MetS: metabolic syndromes
MCI: mild cognitive impairment
MS: multiple sclerosis
NF&ND: neuroinflammation & neurodegeneration
NAFL: nonalcoholic fatty liver
PD: Parkinson’s disease
PRRs: pattern recognition receptors
PVS: perivascular space
PP: Peyer’s patch
Plvap: plasmalemma vesicle-associated protein
PKA: protein kinase A
Tregs: regulatory T cells
RES: reticuloendothelial system
SAA: serum amyloid A
SHH: sonic hedgehog
SNI: spared nerve injury
TCs: T cells
TJs: tight junctions
TRM: tissue-resident memory
TLRs: Toll-like receptors
TMAO: trimethylamine-N-oxide
VCAM-1: vascular cell adhesion molecule 1
VEGF: vascular endothelial growth factor
Wnt: Wingless/Integrated
